# Expression, function and clinical application of stanniocalcin‐1 in cancer

**DOI:** 10.1111/jcmm.15348

**Published:** 2020-05-29

**Authors:** Fangyu Zhao, Gang Yang, Mengyu Feng, Zhe Cao, Yueze Liu, Jiangdong Qiu, Lei You, Lianfang Zheng, Taiping Zhang, Yupei Zhao

**Affiliations:** ^1^ Department of General Surgery Peking Union Medical College Hospital Chinese Academy of Medical Sciences and Peking Union Medical College Beijing China; ^2^ Department of Nuclear Medicine Peking Union Medical College Hospital Chinese Academy of Medical Sciences and Peking Union Medical College Beijing China; ^3^ Clinical Immunology Center Chinese Academy of Medical Sciences and Peking Union Medical College Beijing China

**Keywords:** biomarker, cancer development, clinical application, stanniocalcin‐1

## Abstract

The glycoprotein stanniocalcin‐1 functions as a regulatory endocrine hormone that maintains the balance of calcium and phosphorus in bony fish and as a paracrine/autocrine factor involved in many physiological/pathological processes in humans, including carcinogenesis. In this review, we provide an overview of (a) the possible mechanisms through which STC1 affects the malignant properties of cancer, (b) transcriptional and post‐transcriptional regulation pathways of STC1 and (c) the potential clinical relevance of STC1 as a cancer biomarker and even a therapeutic target in the future. Exploring the role of STC1 in cancer development may provide a better understanding of the tumorigenesis process in humans and may facilitate finding an effective therapeutic method against cancer.

## 1 INTRODUCTION

Stanniocalcin (STC)‐1 is a glycoprotein first discovered in the corpuscles of Stannius in fish^1^, ^2^ and is believed to regulate serum calcium and phosphate homeostasis in the fish body as an endocrine hormone.^3^ The first mammalian form of STC was discovered in 1996,^4^ which is located on the short arm of chromosome 8 (8p11.2‐p21).^5^ An additional STC family member was subsequently identified,^6^ and they were renamed as STC1 and STC2. The human *STC1* gene is widely expressed in many tissues, but STC1 is normally undetectable in serum,^2^ suggesting that STC1 may act as a paracrine/autocrine factor rather than an endocrine hormone.^7^ STC1 has appeared to change its function during evolution. In mammals, it acts preferentially on phosphate metabolism compared with calcium metabolism. STC1 has been shown to stimulate phosphate reabsorption in the small intestines and proximal tubules of the kidney.^7^ STC1 also participates in many kinds of physiological and pathological processes including pregnancy,^8^, ^9^ lactation,^7^, ^10^ angiogenesis,^11^ bone and muscle development,^12^, ^13^ organogenesis,^14^ cerebral ischaemia,^15^, ^16^, ^17^ ischaemia/reperfusion kidney injury,^18^, ^19^ idiopathic pulmonary fibrosis^20^ and hypertonic stress.^21^ However, its role in cancer has attracted much attention,^5^ and many studies have shown that STC1 promotes tumour cell viability and proliferation, and facilitates solid tumour invasion and metastasis. STC1 might be modulated by HIF‐1 expression under hypoxia condition, thereby initiating expression of several downstream genes. As a result, the Warburg effect will be turned on, reprogramming of tumour metabolism from oxidative to glycolytic metabolism.^22^ What's more, it has been proved that STC1 is involved in multiple cancer‐related signalling pathways, such as NF‐kB,^23^ ERK1/2^24^ and JNK signalling pathways.^25^ Epithelial‐mesenchymal transition (EMT) is a process in which epithelial cells acquire mesenchymal features, associated with tumour initiation, invasion, metastasis and resistance to therapy.^26^ STC1 also participates in EMT process to reshape the tumour microenvironment, promote the initiation of malignant phenotypes.^27^ Clinical data have also revealed that STC1 may have a negative correlation with prognosis, a higher STC1 expression level in tumour tissues was correlated with shorter DFS (disease‐free survival) and OS (overall survival).^28^, ^29^, ^30^ These evidences suggest that STC1 is a potential diagnostic and prognostic marker as well as therapeutic target of cancer. The secretion of STC1 is intermittent and can be stimulated by external stimulus, and thus, the secretion of STC1 is more susceptible to external cytokines related to cancer. While the secretion mode of STC2 is constitutive, which implies STC2 participates the body's normal physiological activity.^5^ So we want to explore the function of STC1 thoroughly for it may be regulated by many cancer‐related factors. Moreover, the researches about STC1 and cancer are more abundant than STC2. Therefore, we will discuss the possible mechanisms through which STC1 interacts with cancer cells and its clinical relevance in this review.

## 2 INTERACTIONS BETWEEN STC1 AND CANCER CELLS

### 2.1 STC1 is aberrantly expressed in tumours

An aberrant expression level of STC1 has been found in many kinds of tumours (Table 1). It is usually overexpressed in most human cancer tissues compared with normal tissues, such as ovarian cancer,^31^, ^32^ hepatocellular carcinoma (HCC),^25^, ^33^ non‐small cell lung cancer,^34^ lung adenocarcinoma,^35^ glioma,^36^, ^37^ thyroid cancer,^38^ fibrosarcoma,^39^ oesophageal squamous cell carcinoma,^28^ laryngeal squamous cell carcinoma (LSCC),^40^ colorectal cancers,^41^, ^42^, ^43^ breast carcinoma,^44^, ^45^ leukaemia^46^, ^47^ and gastric cancer.^48^ However, Guo et al and Pan et al observed decreases of STC1 expression in cervical cancer tissues, especial at stages II and III, compared with adjacent normal tissues and stage I cervical cancer tissues.^49^, ^50^ The reason is that NF‐κB pathway is activated in cervical cancer, leading to an increased level of phospho‐P65 and a corresponding decreased level of non‐phosphorylated P65 protein. As Guo et al^49^ reported, NF‐κB p65 protein directly bound to STC1 promoter and activated the expression of STC1 in cervical cancer cells, thus the decreased non‐phosphorylated p65 protein level then leads to the reduced expression of STC1, the phenomenon we observed in tumour tissues.

**TABLE 1:** Expression levels and functions of STC1 in different kinds of cancers

Type of cancer	STC1 expression level	STC1 Effects on tumour	Source
Cervical cancer	↑	Inhibit cell proliferation, migration and invasion	43, 44
Colorectal cancer	↑	Stimulate migration and invasion	35‐37
Ovarian cancer	↑	Increase cell proliferation and migration	22
Ovarian cancer	↓	Inhibit cell proliferation, promote apoptosis	46
Breast cancer (TNBC)	↑	Promote metastasis	50, 51
Breast cancer (ER+)	↑	Increase cell proliferation	52
Breast cancer (BRCA1‐mutant)	↓	Inhibit cell proliferation, promote apoptosis	47
Laryngeal squamous cell carcinoma	↑	Correlated with advanced clinical stage	34
Leukaemia	↑	Promote chemoresistance, marker of MRD (minimal residual disease)	50, 41
Gastric cancer	↑	Associated with more lymph metastasis and advanced clinical stage	42
Non‐small cell lung cancer	↑	Associate with advanced tumour stage and histological subtype	27
Glioma	↑	Associate with high pathological grade	29, 30
Hepatocellular carcinoma	↑	Associate with tumour size (<5 cm in diameter)	24
Hepatocellular carcinoma	↓	Associate with tumour size (>6.3 cm in diameter)	25, 87
Thyroid cancer	↑	Enhance cell proliferation, inhibit cell apoptosis	31
Oesophageal squamous cell carcinoma	↑	Associate with advanced T‐stage	33
Lung adenocarcinoma	↑	Increase cell proliferation, inhibit cell apoptosis	28

Additionally, the correlation between STC1 expression and breast/ovarian cancer is intricate. Some previous studies have found that STC1 expression down‐regulates after loss‐of‐function of BRCA1, which leads to breast and ovarian cancer.^51^, ^52^, ^53^ Because STC1 is critical for normal breast and ovarian physiologies,^54^ and relatively highly expressed in the ovary with expression dramatically increasing during pregnancy and lactation,^55^ the loss of STC expression in tumour cells may be a phenomenon of malignancy because the normal physiological activity has been disrupted. However, this is the opposite view from recent studies showing that STC1 is overexpressed in breast and ovarian cancer tissues compared normal tissues.^31^, ^56^ In this regard, the tumour source and/or variability are the likely causes of this discrepancy. Triple‐negative breast cancer (TNBC) tends to have a very high STC1 level compared to normal tissues,^56^, ^57^ and ER‐positive tumours also possess a relatively higher STC1 expression level than normal,^58^, ^59^ whereas STC1 may be absent in BRCA1‐mutant breast tumours.^53^ Such evidence implies that STC1 may have a tumour‐promoting function in most cases, but possesses a tumour‐suppressing trait in a few cases.

### 2.2 STC1 acts via an autocrine signalling pathway in tumours

It has been reported that STC1 acts in a paracrine manner to regulate corpora lutea functions,^60^ but STC1 might perform its oncogenic function through an autocrine manner. Mccudden et al^58^ showed that STC1 and its receptor were colocalized in 91% of examined human breast cancer biopsies (53/58) by immunocytochemistry and in situ ligand and binding staining, and all cancerous cells were positively stained for both the ligand and receptor. This pattern is analogous with that in the kidney where an autocrine signalling pathway has been identified in the collecting ducts, whose cells express STC and possess high affinity STC receptors.^61^ These results suggest STC1 appears to be operating via an autocrine loop in the malignant mammary gland, which differs from its paracrine signalling pathways in tissues such as ovaries.

### 2.3 STC1 is regulated by a wide range of factors related to tumours

**FIGURE 1:**
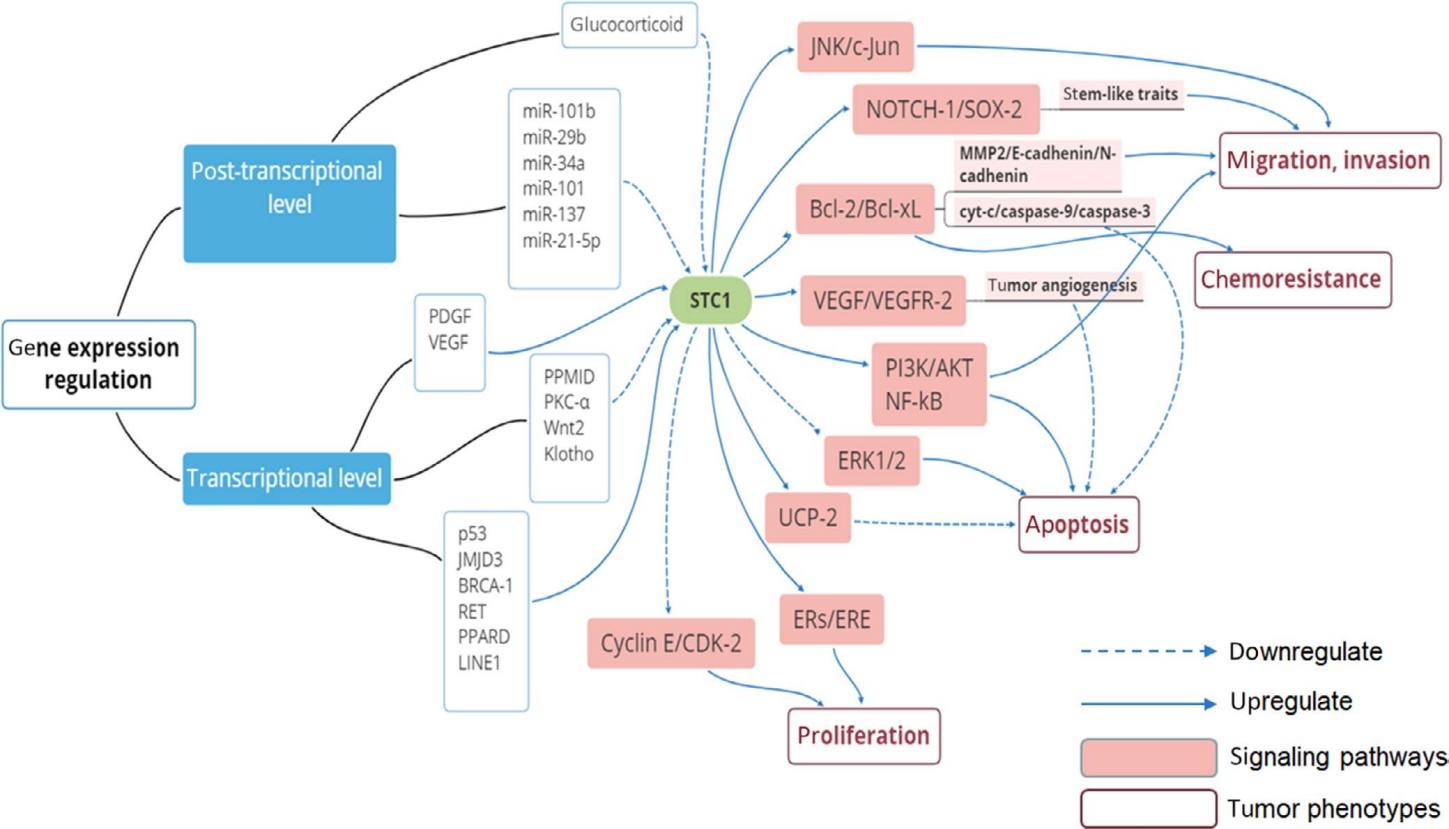
Regulation networks of STC1. STC1 is regulated by many proteins, and it can affect several signalling pathways to modulate tumour phenotypes

The *STC1* gene may be the downstream target of several genes and proteins related to cancers (Figure 1), including p53,^62^ BRCA1,^53^ RET,^63^ Wnt2,^64^ JMJD3,^65^ PPM1D,^66^ PKCα,^67^ PPARD,^68^ LINE‐1,^69^ IGF‐1^70^ and CAIX.^57^

The *STC1* gene promoter is repressed by HDAC, a p53‐dependent histone deacetylase^71^ and a repressor complex formed by transcription factor Sp1 and Rb inhibits *STC1* gene transcription.^72^ It has been reported that one of the key proteins that mediates p53‐induced apoptosis, NF‐kB, also regulates *STC1* gene transcription.^23^ Based on these data, Ching et al^62^ showed that, instead of direct involvement of transactivation of *STC1* gene expression, p53 actives the NF‐kB signalling pathway and inhibits the activity of HDAC then increases the levels of histone H3 acetylation to elevate *STC1* gene expression. Another study confirmed that induction of *STC1* expression in an apoptotic human nasopharyngeal cancer cell line (CNE2) is mediated by activation of p53.^73^ Welcsh et al^53^ described the absence of BRCA1 expression in primary breast tumour cells without expression of STC1 and that BRCA‐1‐induced cells had a much higher expression level of STC1 compared with control cells. They confirmed that BRCA1 is a stimulator of *STC1* expression by oligonucleotide array‐based expression profiling. RET, an oncogene responsible for multiple endocrine neoplasia (MEN) type 2A and 2B that develops into medullary thyroid carcinoma and pheochromocytoma, has been proven to induce STC1 expression, which is in line with immunohistochemistry results indicating that STC1 is highly expressed in specimens of MEN2B‐MTC.^63^ JMJD3, a kind of histone demethylase that correlates with melanoma growth and metastasis, also up‐regulates STC1 expression.^65^ In addition, an increased STC1 expression level after protein phosphatase magnesium‐dependent 1 delta (PPM1D) silencing indicated that *STC1* was affected by PPM1D, a protein overexpressed in various cancer cell lines, which has been associated with poor prognoses of cancers.^66^ Moreover, STC1 expression is suppressed by PKCα (protein kinase Cα), a highly expressed protein in breast cancers. After depleting PKCα expression by siRNA, the STC1 expression level is particularly increased.^67^ Klein et al^64^ demonstrated that *STC1* is a target gene of Wnt signalling and can be down‐regulated by Wnt2, which acts as an angiogenic growth factor and differentiation factor in vascular development during cancer progression. In addition, VEGF treatment stimulates *STC1* gene transcription and increases its mRNA levels.^74^, ^75^ Combined with the aforementioned evidence that STC1 promotes the expression of VEGF, these data suggest positive feedback regulation between STC‐1 and VEGF.

Some miRNAs have also been reported to regulate *STC1* gene expression post‐transcriptionally. Sakata et al reported *STC1* as a target of miR‐101b by several experiments in which miR‐101b bound to three sites in the 3′‐UTR of the *STC1* gene, thereby blocking the translation of STC1 mRNA, but not inducing mRNA degradation.^76^ Sakata et al also reported that miR‐29b, miR‐34a, miR‐101 and miR‐137 have predicted binding sites in STC1 mRNA and down‐regulate both the mRNA level and protein concentration.^77^ Similarly, Lu et al^78^ found that *STC1* is a potential target gene of miR‐21‐5p. Furthermore, Groves et al reported that glucocorticoid potently inhibits *STC1* gene expression through a post‐transcriptional mechanism. Hydrocortisone and dexamethasone down‐regulate the STC1 expression level dramatically in several kinds of mouse and human tumour cell lines, and this effect is antagonized by activation of the cAMP signalling pathway. It is possible that cAMP inhibits the activity of glucocorticoid‐induced protein(s) that destabilize STC mRNA, thereby preserving STC1 expression.^79^

These factors regulate STC1 expression differently and antagonistically. STC1 expression is regulated by a complex network in which factors that promote tumorigenesis can either induce or suppress STC1 expression, and the same mechanism operates for factors that inhibit tumorigenesis. Such examples indicate that STC1 is an intermediate modulated by many tumour‐related factors rather than a determining factor in tumorigenesis, causing it plays distinct functions in different kinds of cancers.

### 2.4 Effect of STC1 on apoptosis in cancer

STC1 has been identified as a stimulator of mitochondrial respiration because it stimulates activity of the mitochondrial electron transport chain and calcium transport in a concentration‐dependent manner. Therefore, STC1 is likely to be an anti‐apoptosis and oncogenic factor because it provides additional energy for tumour cell growth.^80^ However, it is now believed that STC1 performs its cytoprotective function mainly through a hypoxic signalling pathway. It has been observed that STC1 expression is elevated under hypoxic conditions in tumours. For example, Yeung et al confirmed that hypoxia stimulates STC1 gene expression in various human cancer cells including colon carcinomas, nasopharyngeal cancer cell lines (CNE‐2, HONE‐1 and HK‐1), and ovarian cancer cell lines (CaOV3, OVCAR3 and SKOV3).^22^ This effect is believed to be mediated by hypoxia inducible factor (HIF)‐1α, a key transcriptional factor in the hypoxic response. Hypoxia stimulates the expression of HIFs and thus induces overexpression of stanniocalcin‐1.^81^ Subsequently, a study by Law et al using a normoxic human nasopharyngeal cancer cell line (CNE2) confirmed that the STC1 gene has an authentic hypoxia response element (HRE) motif located at the upstream region between −2322 and −2335.^82^ This HIF‐HRE combination requires recruitment of p300, a transcription coactivator.^22^ CAIX, a pH‐regulating enzyme that plays a key role in maintaining alkaline intracellular pH under hypoxic conditions, is also indispensable for STC1 expression and functions as a mediator during hypoxia‐induced STC1 expression. CAIX is up‐regulated under hypoxic conditions as a direct transcriptional target of HIF‐1 and then elevates STC1 expression.^57^

**FIGURE 2:**
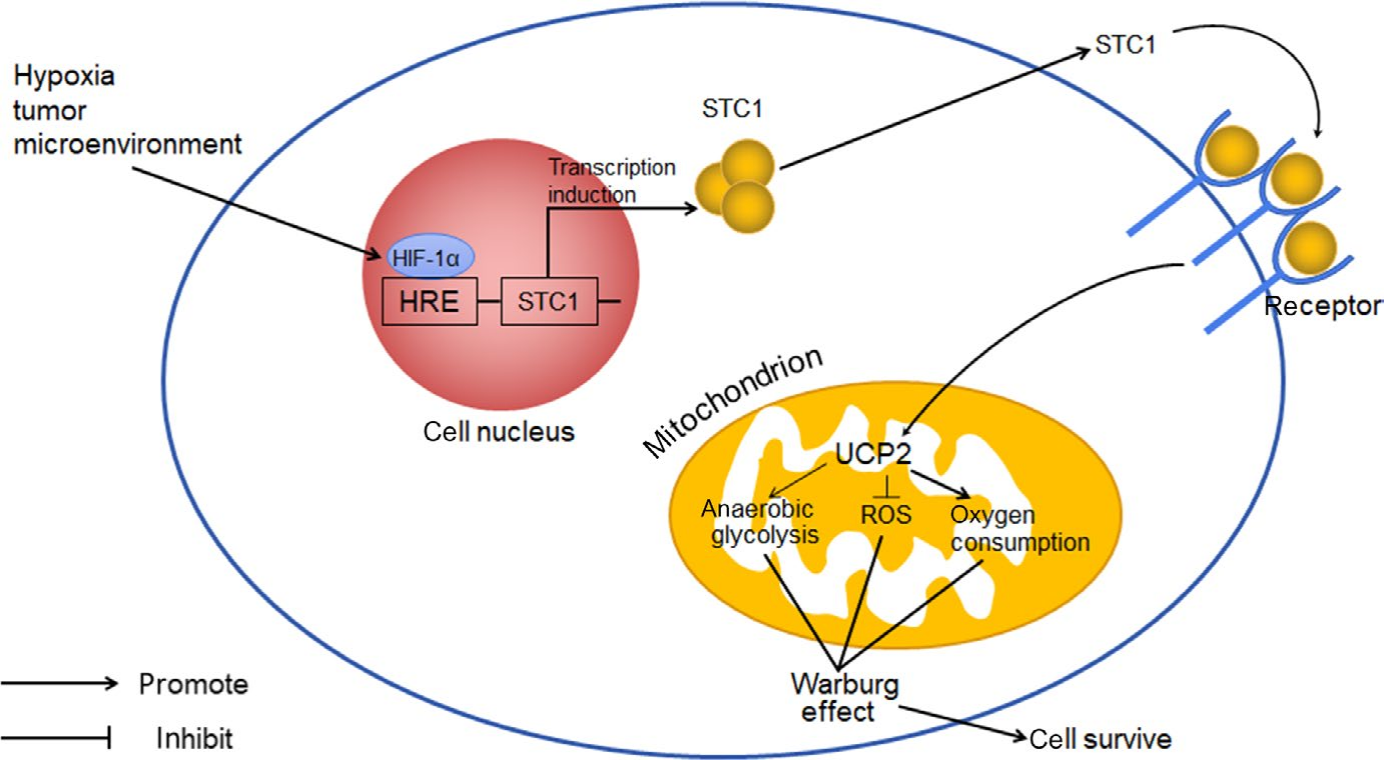
STC1 and hypoxia. HIF, hypoxia inducible factor; HRE, hypoxia response element; UCP, uncoupling protein; ROS, reactive oxygen species

It has been well demonstrated that STC1 enhances tolerance to hypoxia in tumour cells and thus has an anti‐apoptosis effect on tumour cells (Figure 2). In cancer cells, the metabolism changes from aerobic respiration to glycolysis under hypoxia, a much more anaerobic respiratory mode.^83^ During this process, STC1 up‐regulates the expression level of UCP2 (uncoupling protein 2) and decreases the mitochondrial membrane potential.^84^ As a result, the lactate production for anaerobic glycolysis has been increased (also known as the Warburg effect), which promotes the growth of cancers. Oxidative phosphorylation is normally coupled to superoxide generation, such as ROS, which are reactive species that cause oxidative damage to cellular biomolecules and play a major role in pathophysiological processes.^85^ The Warburg effect shifts mitochondrial respiration to a much more glycolytic metabolic profile, reducing ROS generation.^86^ Taken together, STC1 reduces the generation of ROS, diminishes oxidative and ER stresses,^87^ and makes tumours resistant to ROS,^86^ thereby facilitating cancer cells to survive and proliferate.^88^ In addition, Wang et al reported that STC1 promotes the expression of Bcl‐2, leading to decreased caspase‐9/‐3‐dependent cell death under hypoxia.^89^

It is noteworthy that there are also some studies supporting the notion that STC1 can also be a proapoptotic factor. Despite Nguyen et al reporting a high expression level of STC1 under oxidative stress in accordance with the aforementioned studies, they found that STC1 reduces survival and promote apoptosis by down‐regulating a prosurvival signalling pathway, ERK1/2.^24^ In addition, Pan et al reported that STC1 promotes apoptosis via NF‐κB phospho‐p65 (Ser536) by PI3K/AKT, IκBα and IKK signalling in cervical cancer cells.^50^ Yeung *et al* also found a proapoptotic effect of STC1 in hepatocellular carcinoma cell lines Hep3B and MHCC‐97L through up‐regulating proapoptotic genes interleukin‐12 and NOD‐like receptor family, pyrin domain‐containing 3, thereby slowing down the process of carcinogenesis.^90^ Moreover, trichostatin A [TSA; a histone deacetylase (HDAC) inhibitor], as one of the most common treatment methods to induce apoptosis in human cancer cells, induces overexpression of STC1 through activation of p53 in cancer cells.^62^ In addition, TSA‐induced apoptotic processes were found to be significantly reduced by silencing STC1 expression, which supports the notion that STC1 is a proapoptotic factor.^23^

The contradictory roles of STC1 in apoptosis may be because STC1 plays different roles on different pathways related to apoptosis. And the particular role of STC1 may be dependent on the extent of cellular stress.^62^

### 2.5 Effect of STC1 on cancer cell proliferation

STC1 is believed to promote the proliferation of several kinds of tumour cells. For example, recently, Dai et al found that STC1 is involved in the interaction of Klotho and tumour progression. Klotho, a newly found anti‐ageing gene, reduces proliferation of thyroid cancer cell lines FTC133 and FTC238 and enhances apoptosis. A high level of Klotho was found to be associated with a low level of STC1 in both cell lines. Thus, they speculated that Klotho may inhibit thyroid tumour cell proliferation by inhibiting the expression level of STC1. Furthermore, they verified that recombinant STC1 markedly enhanced cell proliferation. However, the detailed molecular mechanism of Klotho‐mediated cell proliferation and apoptosis remains unclear, and Klotho‐induced apoptosis was only tested in thyroid cancer cell lines FTC133 and FTC238. Therefore, further validation is required in other types of thyroid cancer cell lines and animal models.^91^ Furthermore, in a study aimed to elucidate the function of STC1 in prostate carcinoma, Bai et al found that knock‐down of STC1 decreased the proliferation of prostate carcinoma cell lines DU145 and LNCaP2. During this process, the expression levels of cell cycle‐related proteins, cyclin E1/CDK2, were elevated. In contrast, overexpression of STC1 in normal prostate cell line RWPE‐1 and xenografted tumours promotes cell growth.^92^ Similarly, Ma et al described a consistent result that STC1 promotes cell cycle progression, accelerates G1/S transition through elevating the expression of cyclin D1, Cdk4 and Cdk6, and suppressing the expression of p21.^93^ Therefore, STC1 promotes cancer cell proliferation via a novel mechanism through which several cyclins and CDKs are recruited to regulate the cell cycle positively.

However, the effect of STC1 on cell proliferation in breast cancer depends on the subtype. In 4T1ch9 and MDA‐MB‐231HM‐luc cells, which are triple‐negative (ER‐/PR‐/HER2‐) breast cancer cell lines, STC1 expression has no effect on cell proliferation.^94^ Conversely, in T47D cells, a luminal (ER+/PR+) breast cancer line, reduction of STC1 expression reduces in vitro cell proliferation.^51^ This phenomenon can be explained by a proliferation‐promoting effect of STC1 on ER + breast cancer cells, which is dependent on the ER, because the STC1 gene is coexpressed with the ER. STC1 and its receptor were found to be present in all oestrogen receptor‐positive samples (30/30),^58^ which was in agreement with the finding that STC1 and STC2 proteins and mRNAs are highly correlated with ER levels.^95^ ERs function as transcription factors when they are bound to oestrogens. Upon hormone binding, ERs bind to DNA with high affinity at specific sites that are termed oestrogen response elements (EREs) to exert the physiological function of oestrogens. In addition to signalling through EREs, there are alternative, non‐classical ER transcriptional response pathways through which the ensuing expression of ER‐regulated genes results in cell proliferation.^96^, ^97^

### 2.6 Effect of STC1 on invasion and metastasis of cancer cells

STC1 promotes tumour metastasis via activation of PI3K/Akt and JNK signalling pathways. Li et al showed that the treatment with recombinant human STC1 significantly increases the invasiveness of TNBC cells, which was mediated by phosphorylation of JNK/c‐Jun leading to up‐regulation of matrix metalloproteinase 9.^56^ Consistent with this result, secretory STC1 enhances the metastatic potential of HCC via the JNK signalling pathway.^25^ Histone methylation is an important epigenetic mark that regulates gene expression. Aberrant histone methylation patterns caused by deregulated histone demethylases have been associated with carcinogenesis. The histone H3 lysine 27 (H3K27) demethylase JMJD3 up‐regulates STC1 expression, activates NF‐κB and PI3K pathways and promotes distant metastasis of melanoma, indicating that a high STC1 expression level is related to metastasis.^65^ Analysis of transcriptome profiling identified STC1 as a peroxisome proliferator‐activated receptor‐δ (PPARD) target gene. Expression of STC1 is up‐regulated during the metastasis process induced by overexpression of PPARD in cancer cells, indicating a pro‐metastasis effect of STC1 on cancer.^68^ In addition, Li et al pointed out that STC1 interacts with NOTCH1 to activate the SOX2 signalling pathway, augmenting the stem‐like traits of GBM cells, and Sakata et al^77^ found STC1 is a promoting factor of metastasis in GBM. Taken together, we speculated STC1 augments the stem‐like traits of GBM cells and thus enhancing tumourigenicity and distal metastasis, since stem cell‐like properties of cancer cells play a major role in metastasis. Furthermore, expression of STC1 is elevated, which functions as a mediator during the metastasis process induced by platelet‐derived growth factor (PDGF), a major functional determinant of cancer‐associated fibroblasts (CAFs). Elevated expression of PDGF receptors on stromal CAFs is associated with metastasis and a poor prognosis of colorectal cancer. Taken together, PDGF‐stimulated fibroblasts increase the migration and invasion of colorectal cancer cells in an STC1‐dependent manner.^98^ Carbonic anhydrase IX (CAIX) is overexpressed in a variety of solid cancers, including breast cancer, which has been implicated in the migration, invasion and stemness of breast cancer cells. The biological effects caused by inhibition of CAIX reduce invasiveness and the self‐renewal capacity by blocking STC1 induction, suggesting that STC1 promotes the invasiveness of breast cancer cells.^57^

### 2.7 STC1 promotes chemoresistance in tumours

The hypoxic tumour microenvironment plays a prominent role in the induction of chemoresistance.^99^ Because STC1 is closely related to hypoxia in the tumour microenvironment, it may be possible that STC1 contributes to the chemoresistance feature of cancers. Although previous studies have not found a relationship between STC1 expression and chemoresistance,^28^ recent studies have implied a connection between STC1 and chemoresistance in leukaemia because the STC1 expression level was higher in patients whom chemotherapy was unsuccessful compared with responsive patients.^46^, ^47^ Wang et al also found that, under hypoxic conditions, STC1 promotes chemoresistance in gastric cancer patients, probably via up‐regulation of Bcl‐2, a well‐characterized anti‐apoptotic and EMT‐related protein.^89^

### 2.8 STC1 contributes to the tumour‐supporting microenvironment

The tumour microenvironment plays an important role in tumour initiation, progression, metastasis and chemoresistance.^100^ A remarkable feature of the tumour microenvironment is the hypoxic condition induced by rapid proliferation and relatively insufficient vascularization of the tumour mass.^101^, ^102^ STC1 promotes tumour neoangiogenesis that in turn changes the tumour microenvironment. The new vascular system enables tumour cells to obtain enough oxygen and nutrients for survival and proliferation and promotes distal metastasis.

STC1 increases both the mRNA and protein levels of eNOS, VEGF and VEGFR2, and stimulates the VEGF signalling pathway, which subsequently enhances tumour angiogenesis. This promotion effect of STC1 on the expression of VEGF depends on the activation of PKCbII and ERK1/2 pathways.^11^, ^103^ It has been elucidated that stromal cells are recruited to constitute tumorigenic microenvironments and can significantly influence cancer phenotypes.^104^ Fibroblasts and mesenchymal stem cells are important stromal cells that participate in hypoxia‐induced STC1 expression. Fibroblasts can be activated by PDGF, which affect the tumour microenvironment and promote tumourigenicity. STC1 was identified as a fibroblast‐secreted protein^105^ and functions as a mediator of the PDGF receptor and fibroblasts to promote tumour growth and metastasis of colorectal cancer cells.^27^, ^98^ Multipotent human mesenchymal stroma/stem cells (MSCs) are recruited in the tumour microenvironment during carcinogenesis and are believed to support tumour growth for repairing tissue injuries and inflammatory processes caused by tumours.^106^, ^107^ Several studies have found that the supportive effect of MSCs on tumour cells may be partially due to suppression of apoptosis induced by ROS and enhancing the Warburg effect by secreting STC1.^86^, ^108^

## 3 CLINICAL SIGNIFICANCE OF STC1

### 3.1 STC1 serves as a biomarker for cancer diagnosis and early detection

STC1 is highly expressed in various kinds of tumours at both mRNA and protein levels in the blood and tumour tissues of cancer patients compared with patients with benign diseases and healthy individuals, indicating that it can serve as a potential biomarker for cancer diagnosis.^29^ For example, 12 cases out of 15 showed higher expression of STC1 mRNA in oesophageal squamous cell carcinoma tissue compared with normal counterparts. Furthermore, immunohistochemistry showed a high level of STC1 protein in 38.9% (89/229) of patients, mainly localized in the cytoplasm of tumour cells.^28^ Moreover, Arigami et al found that the relative numbers of STC1 mRNA copies were significantly higher in gastric cancer cell lines and in blood specimens from patients with gastric cancer than in blood specimens from healthy volunteers (*P* = .0001 and *P* = .003, respectively). They demonstrated that 65 of 93 (69.9%) patients were positive for STC1 by RT‐PCR analysis. This result indicated superiority to conventional tumour markers CEA and CA‐199 whose positive rates in the same population were 26.9 (25/93) and 24.7% (23/93), respectively. Receiver operating characteristics were used to describe the diagnostic specificity and sensitivity of serum STCs to discriminate patients with gastric cancer from healthy volunteers, and the values were 69.9 and 71.4%, respectively.^29^ Fang *et al* also reported supporting experimental results.^48^

### 3.2 Relationship between STC1 expression and clinicopathological parameters

Elevated expression of STC1 is significantly associated with the tumour grade, size, invasion and metastasis. For example, higher levels of circulating STC1 mRNA in serum were associated with more advanced tumour stages. The expression level of STC1 was significantly higher in the advanced pathological tumour stages of oesophageal squamous cell carcinoma (*P* = .019),^28^ LSCC (*P* = .083),^40^ gastric cancer (*P* = .013),^29^ glioma (*P* < .001),^37^ non‐small cell lung cancer (*P* = .018)^34^ and clear cell renal cell carcinoma (*P* = .008).^93^ In addition, the presence of STC1 expression was significantly correlated with the depth of tumour invasion in gastric cancer (*P* = .032)^29^ and thyroid‐cartilage invasion in laryngeal squamous cell carcinoma (*P* = .086).^40^ Ma et al designed a study focused on clear cell renal cell carcinoma (ccRCC) and found that the STC1 mRNA level in tumour tissues was positively correlated with the average tumour diameter.^93^ In line with this result, a high serum STC1 level was correlated with a larger tumour size in hepatocellular cancer (HCC) patients (*P* = .019, mostly <5 cm).^25^ However, Yeung et al reported a negative correlation of STC1 expression with the HCC tumour size in large tumours (>6.3 cm in diameter). They found that tumours with higher expression levels of STC1 were significantly smaller than those with lower levels of expression (*P* = .008). Although STC1 plays a pro‐oncogenic role in tumour progression, the counteracting effects STC1 on the pro‐inflammatory effects of IL6 and IL8 might slow down the process of carcinogenesis as the tumour grows.^33^, ^90^

STC1 expression levels were elevated to a greater extent in ccRCC tissues and associated with distant metastasis, but only T1 stage tumours exhibited a statistically significant difference (*P* = .021) in STC1 expression compared with other tumour stages, suggesting that STC1 is associated with metastasis of early stage ccRCC.^93^ STC1 also promotes metastasis of gastric cancer, Fang et al^48^ found out that a high STC1 expression level in GC tissues was associated with more lymph metastasis in GC patients (*P* = .029). Breast cancer patients with higher STC1 expression levels in tumour tissues were more likely to have lung metastasis than those who had lower levels (*P* = .02).^94^ Furthermore, the serum level of STC1 mRNA serves as a molecular marker for micrometastases of various human cancers because STC1 mRNA might be useful to detect cancer cells in blood.^109^ For example, an interesting study showed that STC1 is a detectable molecular marker for occult breast cancer with metastasis in the blood and bone marrow.^45^, ^110^ Based on these studies, we speculate that overexpression of STC1 is positively correlated with an advanced tumour grade, larger tumour size and deeper tumour invasion with much more distant metastasis.

### 3.3 STC1 may be a potential prognostic marker for cancer

The STC1 expression level has a close connection with the prognosis of cancer patients. Many studies have shown that overexpression of STC1 with high levels of mRNA and protein in serum and cancer tissues compared with normal counterparts correlates with a poor prognosis of patients. STC1 protein level in tumour tissues has been shown to be associated with much more lymph and distant metastases, stronger invasiveness of the tumour and poorer 5‐year disease‐free survival (or 3‐year progression‐free survival) and the overall survival rate in many kinds of tumours such as ESCC,^28^ glioma^37^ and gastric cancer.^48^ Shirakawa et al^28^ showed that overexpression of STC1 mRNA and protein was significantly correlated with a poor prognosis [overall survival (*P* < .0006) and disease‐free survival (*P* < .0002)] of ESCC patients who had undergone curative surgery. In basal‐type breast cancer patients, high expression of STC‐1 protein in tissues indicated poorer relapse‐free (*P* = .044) and overall survival (*P* = .0054) compared with low expression.^56^ Another study also confirmed a significant association between high STC1 expression and worse OS (*P* = .025) and RFS (*P* = .0007) of patients with basal subtype breast cancer. The association was even stronger when a subgroup of basal‐type BC cases with TP53 mutations was analysed separately, but no such correlations were found for luminal A and HER2 + subtypes.^57^ Furthermore, STC1 may potentiate late breast cancer recurrence because the expression level of STC1 in primary breast tumours was strongly associated with late versus early recurrence at 6‐10 years following diagnosis among ER‐positive, tamoxifen‐treated patients (OR = 2.70, 95% CI: 1.22, 5.98).^111^ TNBC (triple‐negative breast cancer), a subtype of breast cancer, accounts for 15%–20% of breast cancer cases, which is more aggressive and has a poorer prognosis than other subtypes. A study showed that the level of STC1 expression was markedly higher in TNBC cells than in non‐TNBC cells.^56^ Therefore, a much higher level of STC1 in breast cancer patients may indicate a more invasive subtype and worse prognosis. These findings may facilitate development of new methods to diagnose and treat breast cancers using STC1 as a biomarker and treatment target based on the different subtypes of cancer. Nevertheless, the complete function and significance of STC1 in breast cancer is not yet clear and needs further investigation. In addition, STC1 is associated with relapse in leukaemia patients. Patients with AML or ALL, who experienced relapse, had significantly higher STC1 gene expression than those who achieved complete remission (*P* < .001 and *P* = .012, respectively).^46^

Therefore, STC1 is probably an adverse prognostic indicator for patients, but this still needs further verification.

## 4 CONCLUSION

STC1, a glycoprotein hormone first discovered in the corpuscles of Stannius, the endocrine glands on the kidney of bony fish, is a regulatory factor for calcium/phosphate homeostasis and protects the fish against toxic hypercalcemia. It has lost its regulatory function as an endocrine hormone during evolution in mammals and is believed to function as a paracrine/autocrine factor in several physiological and pathological processes. Recent studies also verified that STC1 promotes carcinogenesis in most kinds of cancer, for it prevents cellular apoptosis, promotes cancer cell viability, proliferation and invasiveness.^5^ However, it plays a cancer‐suppressing role in cervical cancer, for it promotes apoptosis, inhibits proliferation and invasion, and enhances the efficacy of chemotherapy.^49^ Thus, its exact role during malignant processes has attracted attention, and many studies to explore its function have been performed thus far. However, the results are sometimes contradictory and the underlying mechanisms of the interaction of STC1 and cancers remain unclear. Therefore, further and deeper investigations are needed to elucidate the full profile of STC1 in carcinogenesis.

It has been reported that STC1 has a distinct clinical significance and is related to the diagnosis, pathological parameters and prognosis of cancer patients. Thus, it may be a potent diagnostic and prognostic marker for patients, which can detect diseases at a much more early stage and evaluate the outcome of patients more precisely. Furthermore, a specific antagonist of STC1 may be a potential therapeutic drug for cancer. Hence, the discovery and application of such therapeutic drugs may be a future goal.

## CONFLICT OF INTEREST

None declared.

## AUTHOR CONTRIBUTIONS

FZ and GY wrote the draft; MF, ZC, YL and JQ created the figures and table; LY and LZ provided guidance for language expression. TZ and YZ made suggestions for revision. All authors approved the final manuscript.
